# Supplementary material for: Vulnerability of short-term memory in a mouse model of Alzheimer’s disease
Source: Nat Commun. 2026 Feb 19;17:2927. doi: 10.1038/s41467-026-69619-2 (PMC13031843; doi:10.1038/s41467-026-69619-2)
Supplement: Supplementary file 1 — Supplementary Information [file 41467_2026_69619_MOESM1_ESM.pdf]

Supplementary Information

Vulnerability of short-term memory in the mouse model of Alzheimer's disease

Chunyue Li[1,†], Xin Wei Chia[1,†], Guozhong Xu[1,†], Lee Fang Ang[1] and Hiroshi Makino[1,2]*

*Correspondence: hmakino@keio.jp

[1]Lee Kong Chian School of Medicine, Nanyang Technological University, Singapore, 308232, Singapore

[2]Department of Physiology, Keio University School of Medicine, Tokyo, 160-8582, Japan

[†]These authors contributed equally.

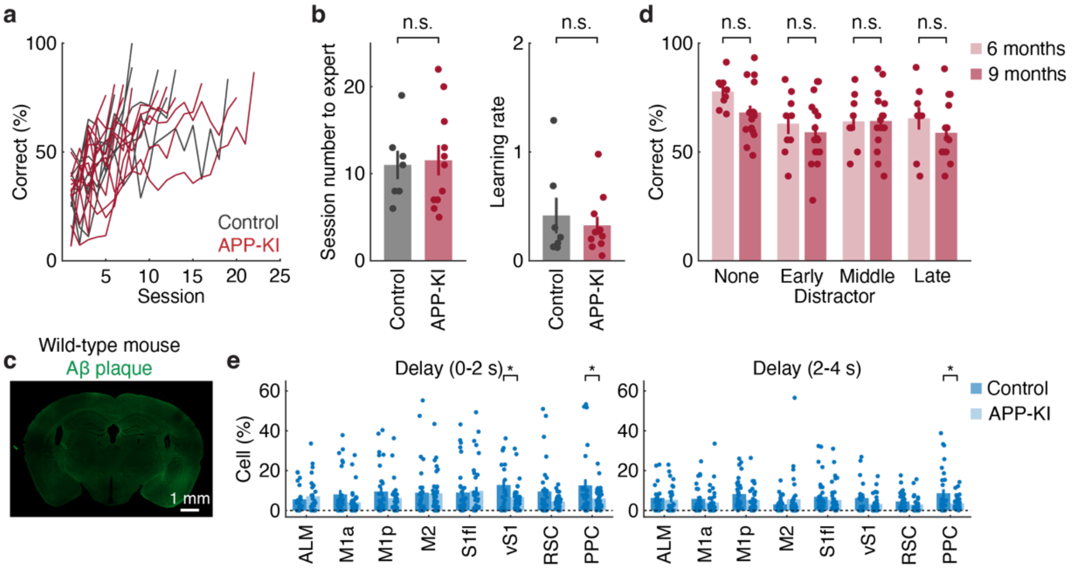

Fig. S1 | Additional analysis of behavior and neural activity during a delayed-response task.

(a) Learning curves of individual control and APP-KI mice (n = 7 and 11 mice for control and APP-KI mice, respectively).

(b) Left. Session numbers required to reach the expert stage in control and APP-KI mice (n.s., P = 0.98, n = 7 and 11 mice for control and APP-KI mice, respectively, two-tailed Wilcoxon rank-sum test). Right. Learning rates in control and APP-KI mice (n.s., P = 1.0, n = 7 and 11 mice for control and APP-KI mice, respectively, two-tailed Wilcoxon rank-sum test). Error bars indicate mean ± SEM.

(c) Example of Aβ staining in a 9-month-old wild-type mouse. No Aβ plaques were detected. Similar images were acquired from 3 mice.

(d) Task performance in non-distractor and distractor trials for 6-month-old and 9-month-old APP-KI mice (n.s., P > 0.05 for all comparisons, n = 9 and 16 sessions for 6-month-old and 9-month-old APP-KI mice, respectively, two-tailed Wilcoxon rank-sum test with an FDR using the Benjamini-Hochberg procedure). Error bars indicate mean ± SEM.

(e) Proportion of neurons exhibiting selectivity during the early (0-2 s) or late (2-4 s) delay period, computed from correct trials (*P < 0.05, control: n = 22, 27, 32, 30, 32, 21, 32, 31 sessions; APP-KI: n = 29, 37, 30, 33, 36, 31, 31, 38 sessions for ALM, M1a, M1p, M2, S1fl, vS1, RSC and PPC, respectively, one-tailed bootstrap with an FDR using the Benjamini-Hochberg procedure). Error bars indicate mean ± SEM.

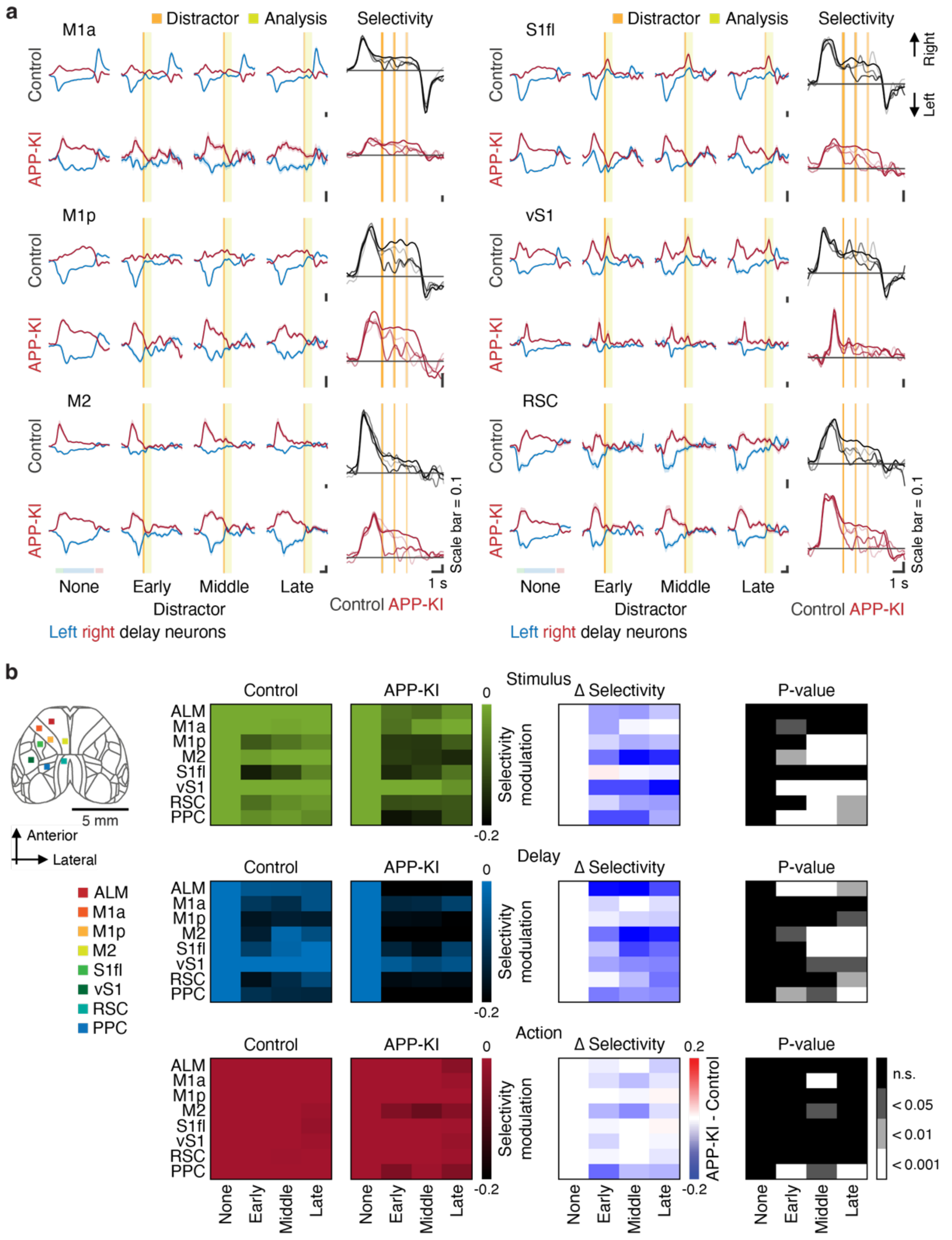

Fig. S2 | Trial-type selective activity modulation in additional regions.

(a) Left. Mean task-related activity differences of delay neurons in six additional regions, with and without distractors, in control and APP-KI mice. Task-related activity differences were computed by subtracting activity during left non-distractor trials from that during right non-distractor trials, resulting in upward and downward deflections for right- and left-preferring delay neurons, respectively. Blue and red traces indicate activity of left- and right-delay neurons, respectively. Orange vertical lines indicate the period when the distractor was present, and green shaded areas mark the 1-s analysis window following each distractor. Right. Mean trial-type selectivity profiles of delay neurons in six additional regions over time for trials with and without distractors in control and APP-KI mice. Trial-type selectivity is represented by the distance between the two activity traces. Shaded areas represent mean ± SEM.

(b) Left. Schematic of the dorsal cortex showing each imaged region. Middle. Region-specific distractor-mediated modulation of trial-type selectivity in control and APP-KI mice, for neurons selective to each trial epoch. Modulation was calculated relative to non-distractor trials within the analysis window (green shaded

areas in **(a)**. Right. Differences between control and APP-KI mice and corresponding p-values (one-tailed bootstrap with an FDR using the Benjamini-Hochberg procedure).

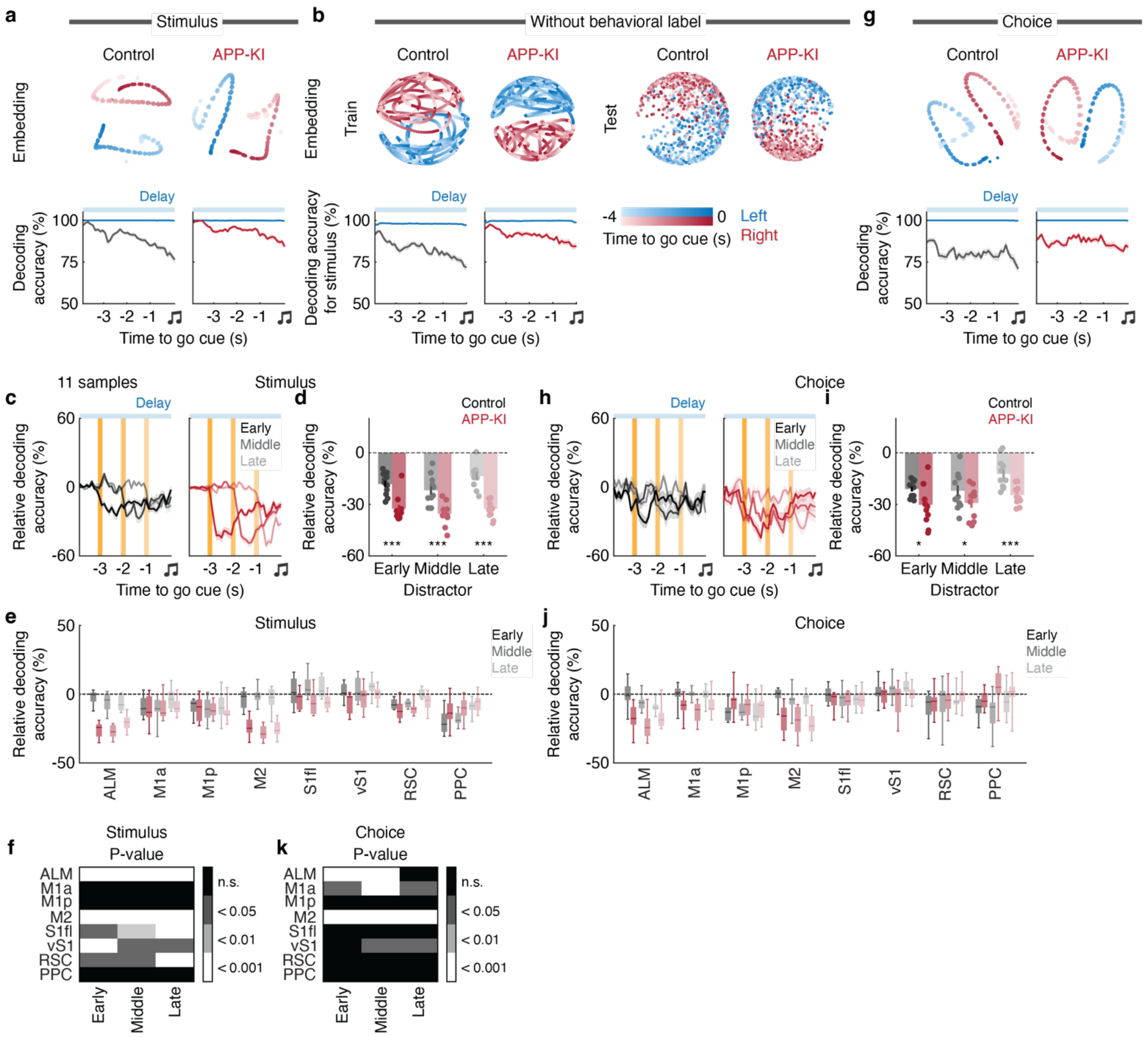

Fig. S3 | Additional analysis of CEBRA.

(a) Top. CEBRA embedding of neural population activity in training datasets using the stimulus as the behavioral label for control and APP-KI mice. Bottom. Moment-by-moment decoding accuracy in test datasets relative to training datasets (blue line) for stimulus selectivity during the delay period in control and APP-KI mice. Shaded areas represent mean ± SEM.

(b) Same as **(a)** but without the behavioral label. CEBRA embeddings and moment-by-moment decoding accuracy for both training and test datasets are shown.

(c) Moment-by-moment decoding accuracy of stimulus selectivity during the delay period in early, middle and late distractor trials for control and APP-KI mice with a sample size of 11 pseudo-mice. Relative decoding accuracy was quantified by subtracting non-distractor trial accuracy from distractor trial accuracy. Orange vertical lines indicate the period when the distractor was present. Shaded areas represent mean ± SEM.

(d) Summary of the decoding results in **(c)** (***$P < 0.001$ for early, middle and late distractors, n = 11 pseudo-mice for control and APP-KI mice, one-tailed bootstrap with an FDR using the Benjamini-Hochberg procedure). Relative decoding accuracy was averaged over a 1-s window following the distractors shown in **(c)**. Error bars indicate mean ± SEM.

(e) Summary of region-specific decoding results with a sample size of 11 pseudo-mice. The analysis window was the same as in **(d)**. The boxes denote the 25th and 75th percentiles, the horizontal lines indicate the median and the whiskers represent the minimum and maximum values.

(f) Corresponding p-values for **(e)** (n = 11 pseudo-mice for control and APP-KI mice, one-tailed bootstrap with an FDR using the Benjamini-Hochberg procedure).
(g) Same as **(a)** but with choice as the behavioral label.
(h) Same as **(c)** but with choice as the behavioral label.
(i) Same as **(d)** but with choice as the behavioral label.
(j) Same as **(e)** but with choice as the behavioral label.
(k) Same as **(f)** but with choice as the behavioral label.

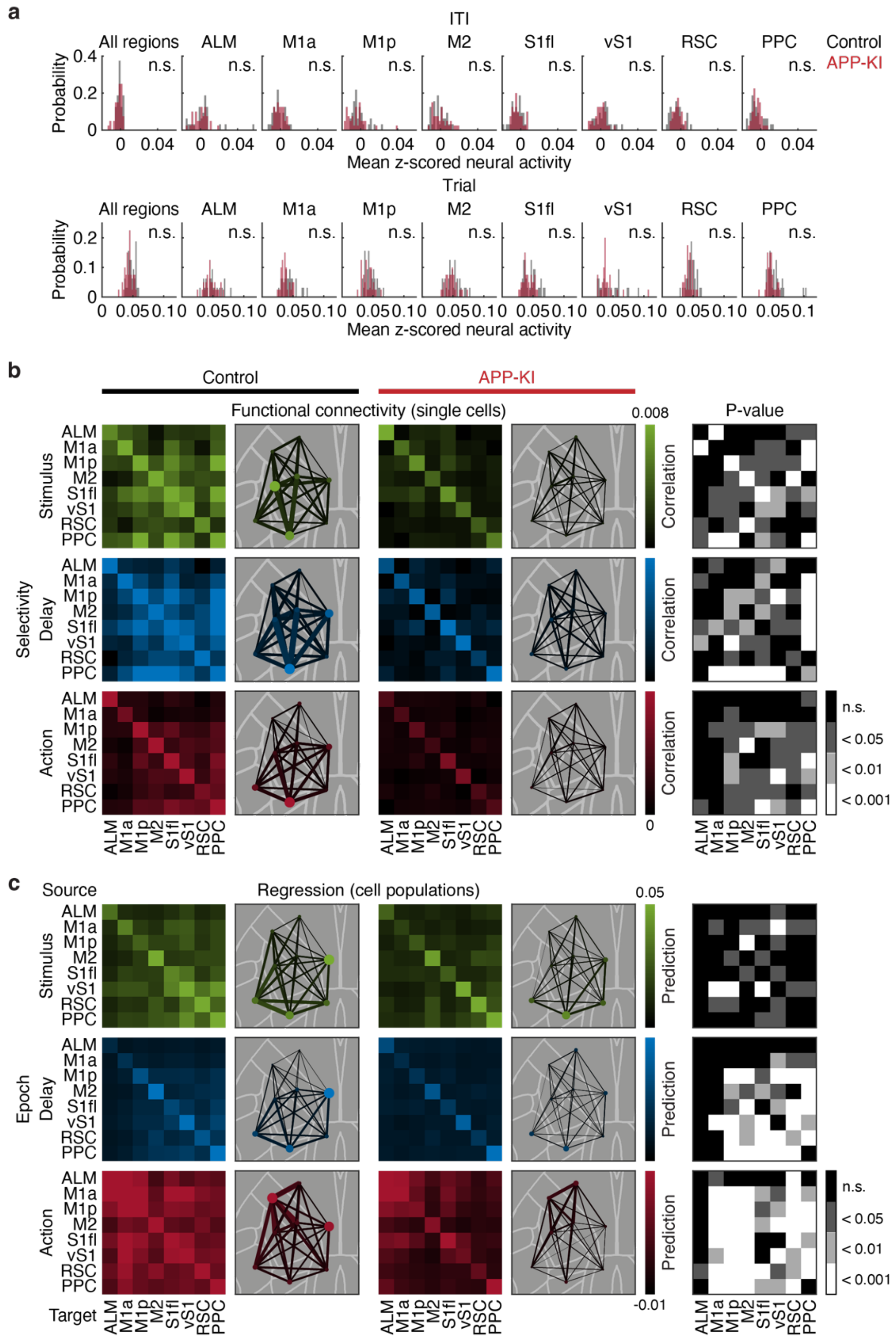

Fig. S4 | Regional communication among neurons with shared task-variable selectivity and regression of population activity across trial epochs.

(a) Probability distributions of mean z-scored activity during the ITI (top) and trial (bottom) in control and APP-KI mice (ITI: n.s., P > 0.05 for all comparisons, control: n = 32, 28, 32, 32, 32, 32, 31, 32, 32 sessions; APP-KI: n = 40, 34, 37, 36, 34, 36, 38, 32, 38 sessions for all regions, ALM, M1a, M1p, M2, S1fl, vS1, RSC and PPC, respectively, two-tailed Kolmogorov-Smirnov test with an FDR using the Benjamini-Hochberg procedure; trial: n.s., P > 0.05 for all comparisons, control: n = 32, 22, 26, 32, 27, 32, 21, 32, 31 sessions; APP-KI: n = 40, 28, 32, 32, 31, 35, 30, 31, 37 sessions for all regions, ALM, M1a, M1p, M2, S1fl, vS1, RSC and PPC, respectively, two-tailed Kolmogorov-Smirnov test with an FDR using the Benjamini-Hochberg procedure).

(b) Left. Regional communication assessed by functional connectivity between pairs of neurons with similar encoding properties during the ITI in control and APP-KI mice. Dot size and color represent intra-regional Pearson correlation coefficients. Line width and color represent inter-regional Pearson correlation coefficients. Right. Corresponding p-values (one-tailed bootstrap with an FDR using the Benjamini-Hochberg procedure).

(c) Left. Regional communication assessed by linear regression of population activity during each task epoch in control and APP-KI mice. Intra- and inter-regional Pearson correlation coefficients are represented as in **(b)**. Right. Corresponding p-values, determined as in **(b)**.

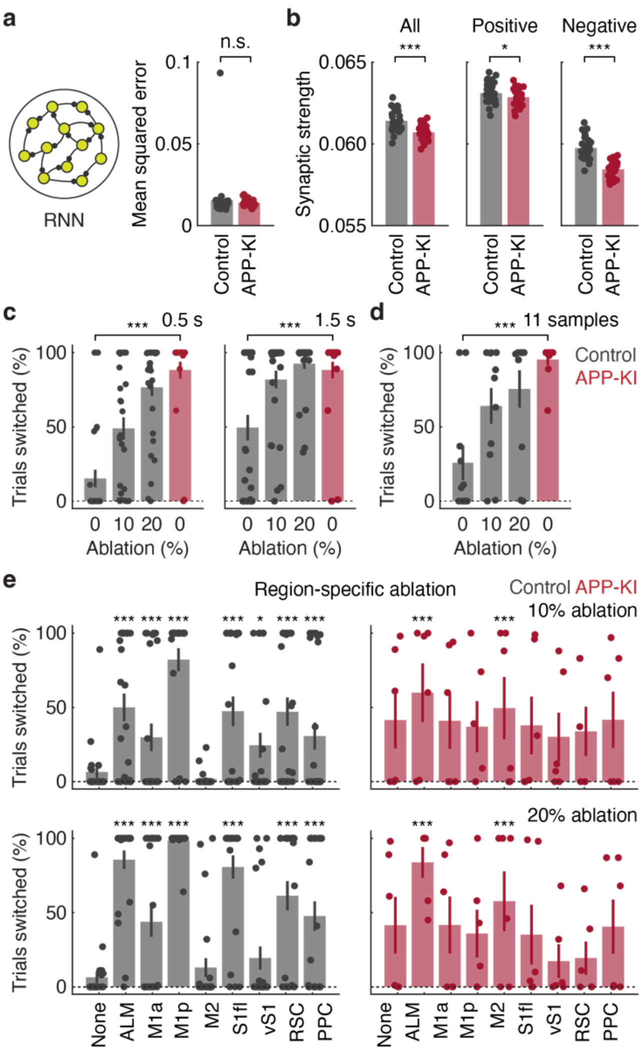

Fig. S5 | Additional analysis of RNNs.

(a) Mean squared errors between the neural activity of cortical neurons and RNN units in control and APP-KI mice (n.s., $P = 0.19$, $n = 30$ RNNs per group, two-tailed Wilcoxon rank-sum test). Error bars indicate mean ± SEM.

(b) Synaptic weights in control and APP-KI RNNs (all: ***$P < 0.001$; positive: * $P < 0.05$; negative: *** $P < 0.001$, $n = 30$ RNNs per group, one-tailed bootstrap). Error bars indicate mean ± SEM.

(c) Left. Mean proportions of trials in which perturbations switched the RNN's choice for control and APP-KI (***$P < 0.001$, $n = 30$ RNNs per group, one-tailed bootstrap) and quantification of network stability as a function of connectivity ablation for control with a 0.5-s analysis window (***$P = 7.88 \times 10^{-16}$, $F(3, 87) = 38.14$, $n = 30$ RNNs, one-way repeated measures ANOVA). Right. Same as left but with a 1.5-s analysis window (control vs. APP-KI: ***$P < 0.001$, $n = 30$ RNNs per group, one-tailed bootstrap; connectivity ablation for control: ***$P = 1.34 \times 10^{-7}$, $F(3, 87) = 14.17$, $n = 30$ RNNs, one-way repeated measures ANOVA). Error bars indicate mean ± SEM.

(d) Same as **(c)** but with a 1-s analysis window and a sample size of 11 RNNs (control vs. APP-KI: ***$P < 0.001$, $n = 11$ RNNs per group, one-tailed bootstrap; connectivity ablation for control: ***$P = 3.43 \times 10^{-5}$, $F(3, 30) = 11.52$, $n = 11$ RNNs, one-way repeated measures ANOVA). Error bars indicate mean ± SEM.

(e) Top. Mean proportions of trials in which perturbations switched the RNN's choice for control and APP-KI mice after region-specific 10% connectivity ablation (*$P < 0.05$, ***$P < 0.001$, $n = 24$ and 6 RNNs for control and APP-KI, respectively, for all regions, paired one-tailed bootstrap with an FDR using the Benjamini-Hochberg procedure). Bottom. Same as top but with 20% connectivity ablation (***$P < 0.001$, $n = 24$ and 6 RNNs for control and APP-KI, respectively, for all regions, paired one-tailed bootstrap with an FDR using the Benjamini-Hochberg procedure). Error bars indicate mean ± SEM.

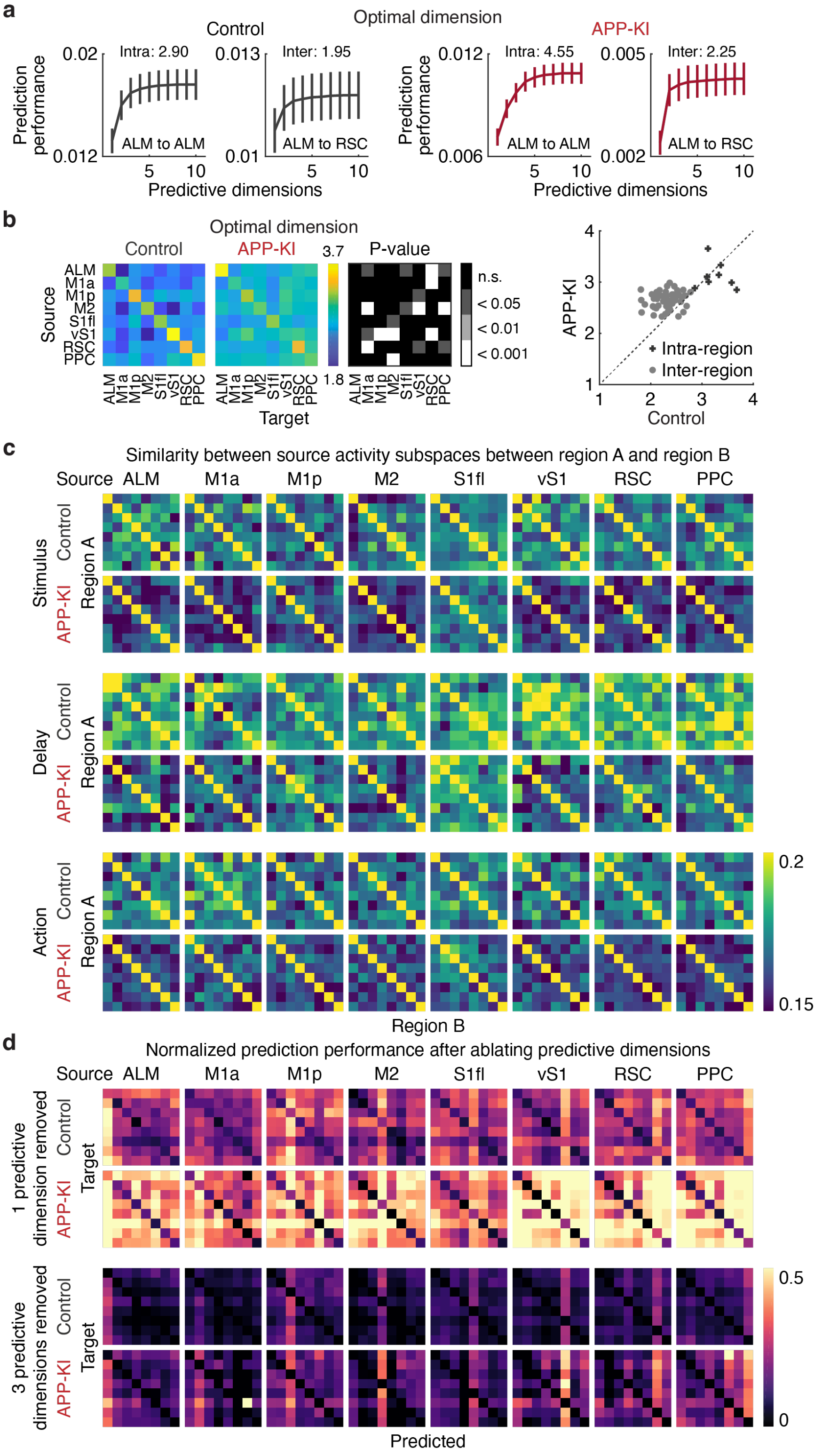

Fig. S6 | Additional analysis of reduced spatial degeneracy in cortico-cortical communication in APP-KI mice.

(a) Examples of reduced-rank regression between source and target regions in control and APP-KI mice. Prediction performance is plotted against the number of predictive dimensions. Optimal dimensionality, indicated in each panel, represents the smallest number of dimensions required to achieve peak prediction performance. Error bars indicate mean ± SEM.

(b) Left. Mean optimal dimension derived from reduced-rank regression in control and APP-KI mice for each source-target pair, with corresponding p-values. Right. Mean optimal dimension of intra- and inter-regional interactions in control and APP-KI mice (main effect: $P = 2.44 \times 10^{-72}$, $F(1, 1976) = 351.64$ for intra- vs.

inter-regions, intra-regional difference: $P = 0.09$; inter-regional difference: $P < 0.001$, two-way ANOVA with Tukey-Kramer post-hoc test).

(c) Subspace similarity between pairs of target regions for each source region across epochs in control and APP-KI mice. Subspace similarity was calculated using the first three dimensions of the communication subspace (Stimulus: main effects: $P = 1.36 \times 10^{-29}$, $F(1, 5125) = 129.24$ for genotype and $P = 1.15 \times 10^{-07}$, $F(7, 5125) = 6.51$ for source regions, two-way ANOVA, ALM: $P < 0.001$; M1a: $P < 0.001$; M1p: $P = 1.00$; M2: $P < 0.01$; S1fl: $P = 0.99$; vS1: $P < 0.001$; RSC: $P < 0.001$; PPC: $P < 0.001$, Tukey-Kramer post-hoc test; delay: main effects: $P = 2.54 \times 10^{-32}$, $F(1, 5125) = 142.04$ for genotype and $P = 1.16 \times 10^{-11}$, $F(7, 5125) = 9.42$ for source regions, two-way ANOVA, ALM: $P < 0.001$; M1a: $P = 0.25$; M1p: $P = 1.00$; M2: $P < 0.01$; S1fl: $P = 1.00$; vS1: $P < 0.001$; RSC: $P < 0.001$; PPC: $P < 0.001$, Tukey-Kramer post-hoc test; action: main effects: $P = 2.48 \times 10^{-19}$, $F(1, 5125) = 81.46$ for genotype and $P = 1.28 \times 10^{-4}$, $F(7, 5125) = 4.20$ for source regions, two-way ANOVA, ALM: $P = 0.05$; M1a: $P < 0.01$; M1p: $P = 0.82$; M2: $P = 0.10$; S1fl: $P = 1.00$; vS1: $P = 0.27$; RSC: $P < 0.01$; PPC: $P < 0.001$, Tukey-Kramer post-hoc test).

(d) Normalized prediction performance across source regions in control and APP-KI mice after predictive dimension ablation. Normalized performance was calculated for source-to-predicted regions after removing one (top) or three (bottom) predictive dimensions from the communication subspace between specific source-target pairs (1 predictive dimension removed: main effects: $P = 1.20 \times 10^{-22}$, $F(1, 12257) = 96.29$ for source–target identity, P below machine precision, $F(1, 12257) = 2039.80$ for target–predicted identity, $P = 8.15 \times 10^{-137}$, $F(1, 12257) = 635.79$ for source–predicted identity and $P = 4.16 \times 10^{-203}$, $F(1, 12257) = 960.53$ for genotype; 3 predictive dimension removed: main effects: $P = 0.012$, $F(1, 12257) = 6.35$ for source–target identity, $P = 4.37 \times 10^{-223}$, $F(1, 12257) = 1060.02$ for target–predicted identity, $P = 7.49 \times 10^{-110}$, $F(1, 12257) = 506.07$ for source–predicted identity and $P = 2.08 \times 10^{-3}$, $F(1, 12257) = 9.48$ for genotype, four-way ANOVA).

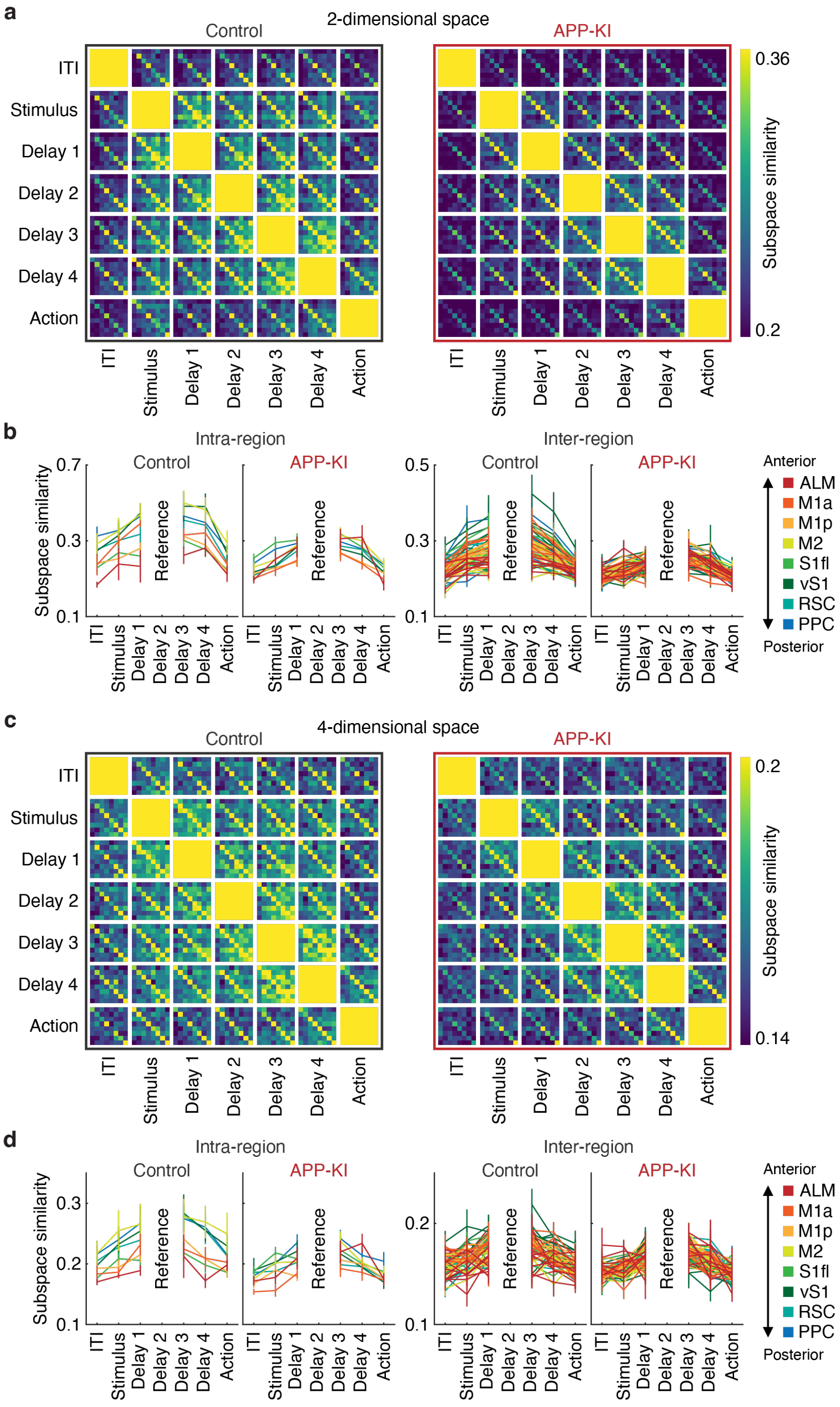

Fig. S7 | Additional analysis of reduced temporal degeneracy in cortico-cortical communication in APP-KI mice.

(a) Region-by-region subspace similarity in two-dimensional space evaluated across seven time points (1-s intervals) in control and APP-KI mice.

(b) Intra-regional and inter-regional two-dimensional subspace similarity with "Delay 2" as the reference in control and APP-KI mice. Subspace similarity was higher near the reference time point (main effects: P below machine precision, $F(1, 11805) = 2785.49$ for intra- vs. inter-regions, $P = 3.34 \times 10^{-196}$, $F(5, 11805) = 190.91$ for trial epoch, $P = 3.11 \times 10^{-45}$, $F(7, 11805) = 32.63$ for source region and $P = 5.36 \times 10^{-84}$, $F(1, 11805) = 383.19$ for genotype, four-way ANOVA). Sample sizes (session numbers) for each source–target

pair and genotype are provided in the Source Data file. Different colors represent individual cortical source regions. Error bars indicate mean ± SEM.

(c) Same as **(a)** but for four-dimensional subspace.

(d) Same as **(b)** but for four-dimensional subspace (main effects: P below machine precision, F(1, 11805) = 1699.84 for intra- vs. inter-regions, $P = 1.82 \times 10^{-103}$, F(5, 11805) = 99.86 for trial epoch, $P = 1.02 \times 10^{-36}$, F(7, 11805) = 26.79 for source region and $P = 4.56 \times 10^{-52}$, F(1, 11805) = 232.81 for genotype, four-way ANOVA).

Table S1. Sex and age of individual control and APP-KI mice at the last imaging session

Mouse	Sex	Age
Control 1	Female	8 months 27 days
Control 2	Male	7 months 11 days
Control 3	Male	8 months 21 days
Control 4	Male	8 months 20 days
Control 5	Male	7 months 13 days
Control 6	Female	9 months 6 days
Control 7	Female	8 months 27 days
APP-KI 1	Male	6 months 25 days
APP-KI 2	Male	8 months 8 days
APP-KI 3	Male	7 months 3 days
APP-KI 4	Female	7 months 5 days
APP-KI 5	Male	6 months 29 days
APP-KI 6	Male	6 months 29 days
APP-KI 7	Male	9 months 26 days
APP-KI 8	Female	7 months 21 days
APP-KI 9	Female	7 months 23 days
APP-KI 10	Female	9 months 23 days
APP-KI 11	Female	9 months 28 days
